# Social position and geriatric syndromes among Swedish older people: a population-based study

**DOI:** 10.1186/s12877-019-1295-8

**Published:** 2019-10-15

**Authors:** C. Rausch, Y. Liang, U. Bültmann, S. E. de Rooij, K. Johnell, L. Laflamme, J. Möller

**Affiliations:** 1 Department of Public Health Sciences, Karolinska Institutet, Widerströmska huset 4:th floor, Tomtebodavägen 18A, 17177 Stockholm, SE Sweden; 2 Department of Health Sciences, Community and Occupational Medicine, University of Groningen, University Medical Center Groningen, Antonius Deusinglaan 1, FA10, 9713 AV Groningen, The Netherlands; 3 Department of Internal Medicine, Center for Geriatric Medicine, University of Groningen, University Medical Center Groningen, Hanzeplein 1, Groningen, 9713 GZ The Netherlands; 4 Department of Medical Epidemiology and Biostatistics, Karolinska Institutet, Nobels väg 12A, Stockholm, 171 77 Sweden

**Keywords:** Geriatric syndromes, Health inequality, Socio-economic status, Social position, Elderly

## Abstract

**Background:**

Older people with a low social position are at higher risk of poor health outcomes compared to those with a higher social position. Whether lower social position also increases the risk of geriatric syndromes (GSs) remains to be determined. This study investigates the association of social position with GSs among older community-dwellers.

**Methods:**

Three consecutive population-based health surveys in 2006, 2010 and 2014 among older community-dwellers (age 65–84 years) in Stockholm County were combined (*n* = 17,612) and linked with Swedish administrative registry information. Social position was assessed using registry information (i.e. education, country of origin and civil status) and by self-reports (i.e. type of housing and financial stress). GSs were assessed by self-reports of the following conditions: insomnia, urinary incontinence, functional decline, falls, depressive disorder, hearing or vision problems. Binomial logistic regression analyses were used to estimate the association between social position and GSs after adjusting for age, sex, health status, health behavior and social stress.

**Results:**

The prevalence of GSs was 70.0%, but varied across GSs and ranged from 1.9% for depression to 39.1% for insomnia. Living in rented accommodation, being born outside the Nordic countries, being widowed or divorced were associated with GS presence. Financial stress was most strongly associated with GSs (adjusted odds ratio, 2.59; 95% CI, 2.13–3.15).

**Conclusion:**

GSs are highly prevalent among older Swedish community-dwellers with wide variations across syndromes and strong association with all measures of social position, most strikingly that of experiencing financial stress.

## Background

Older people typically have both age-dependent conditions, like functional decline, and chronic ones, like cardiovascular diseases [1, 2]. Their co-occurrence challenges outcome specific clinical and public health interventions [2, 3]. Modifiable factors like health behavior and social isolation, but also less modifiable factors like social position play a role in the occurrence of these conditions [1, 4, 5]. While there are several social position measures that have a documented direct association with health for both general and chronic conditions, e.g. level of education or income [1, 4, 6, 7], less is known about how they relate to age-dependent conditions.

One group of age-dependent conditions that warrants closer investigation in that respect is geriatric syndromes (GSs) (e.g. loss of hearing and vision or urinary incontinence) [7–9]. Presence of geriatric syndromes marks older peoples’ frail state. Acting on these syndromes may prevent serious deterioration of existing chronic conditions, as well as the decline in functional disabilities and dependence [2, 10–12]. Time of onset and severity of GSs may be influenced by older people’s health, social stress and health-related behaviors, all of which are closely related to their social position [1, 5, 12–14]. Various measures of social position like education, income, but also civil status have different effect on GSs [1, 8, 13, 15]. However, knowledge on the association between social position and geriatric syndromes is scarce and an investigation into a combination of GSs and measures of social position may provide valuable overview into their relationship. To our knowledge, no previous study has considered this association in the context of Sweden.

GSs have been defined as multifactorial health conditions that visualize and contribute to older peoples underlying frailty [10, 16]. As such they present an increased level of vulnerability for situational challenges due to aging processes and accumulated impairments [10]. While GSs are pluriform and prevalent among community-dwellers [17, 18], they are difficult to manage clinically [5, 10, 17, 19], and put older people at increased risk not only for developing new chronic conditions, but also for hospitalization and/or institutionalization [12]. Various GSs share risk factors and etiologies like older age or impaired mobility [10, 18]. Specific GSs, like falls and urinary incontinence, have been extensively studied and their relation with future health outcomes, such as increased mortality, and decreased quality of life is well established [10, 18, 20, 21]. Other GSs, like the course of functional decline and insomnia have been studied to a lesser extend [10, 22]. However, studies combining several types of GS are rare [12, 18, 19], as consensus on a clear universal definition for GSs is lacking [10, 12]. Studying GSs as a set, rather than individual or specific GSs, helps to get a better understanding of other determinants involved such as health factors and social factors [23]. This approach also helps to study the influence of social position on GSs, which may identify health inequalities in regards to the existence of GSs among older people living in society [10].

In this study, we therefore aim to determine the prevalence of GSs and the associations between social position and GSs among older community-dwellers, taking into account health status, health-related behavior and social stress.

## Methods

### Study design and sample

Data was drawn from three cross-sectional Stockholm County Council Public Health Surveys (i.e. 2006, 2010, and 2014) in Stockholm County, with study design and sample selection previously published (see Additional file 1, [24]). The Public Health Surveys were conducted every four years among approximately 50,000 individuals aged 18–84 years (from 2010, individuals above 84 years were also included).

In the current study, the sample was restricted to people aged 65 to 84 years (*n* = 18,592). The final study population consists of 17,612 participants, excluding those with missing information on GS items (*n* = 980), but including those with missing data on GSs items that do report having at least one GS items present. Among those, the response rates were 74.5% (*n* = 6713), 74.1% (*n* = 7153) and 60.1% (*n* = 4726) in 2006, 2010 and 2014 respectively. Data from self-reports were linked with Swedish registers: the database for health insurance and labor market studies (LISA).

### Measures

Information on GSs and social position, including type of housing and financial stress, was based on self-reported questionnaires. Information on the other measures of social position, including education, civil status and country of origin, was extracted from the LISA register.

### Geriatric syndromes

Seven GSs were assessed [10, 18], including injurious falls in the last six months, urinary incontinence (urinary leakage), functional decline (inability to: run 100 m, as well as walking 100 m or taking stairs), severe hearing problems (despite using hearing aids), severe vision problems (despite the use of glasses), insomnia (light to heavy sleeping difficulties) and signs of depressive disorders (measured by the 12-item General Health Questionnaire [GHQ], Goldberg et al. 1988) [25]. The GHQ-12 is a validated screening device for minor psychiatric conditions. Answering “yes” to at least one GSs, or having a GHQ score > 8 for depressive disorder, defined the presence of a GSs.

### Social position

Social position was assessed by five measures: level of education, civil status, country of birth, type of housing and financial stress. Level of education was categorized as university degree (more than twelve years of education), secondary school (ten to twelve years of education) or primary school ( equal to or less than nine years of education). Civil status was categorized into married, unmarried, divorced and widowed. Country of birth was measured as Sweden, other Nordic countries, other European countries, and the rest of the world. Type of housing was measured as owning an accommodation or housing, rental accommodation or others including second-hand rentals, assisted-communal living and student housing. Financial stress was present when participants indicated to have struggled to buy food, pay bills, rent or things of similar nature in the last 12 months. The measures were separately assessed in their association with GSs and based on previously applied models of social position and inequality in health included the WHO PROGRESS framework [1, 26, 27].

### Confounders

Potential confounders were age, sex, and indicators of the following domains; health status, health behavior and social stress which have previously been shown to related to GSs and social position [4, 13]. The selection of domains and indicators was based on earlier literature on health and life conditions among elderly in Stockholm [5, 28].

Information on age and sex was extracted from the LISA Register. Information on health status, health behavior and social stress was based on self-reports from the Stockholm County Council Public Health Surveys.

Health status was assessed via different proxies: self-reported diagnosis of at least one chronic condition (including diabetes, chronic obstructive pulmonary disease, hypertension, hyperlipidemia, angina pectoris, heart failure, myocardial infarction or stroke), body mass index (BMI) and general self-rated health. The BMI was categorized into obesity (> 29.99 kg/m^2^), overweight (29.99–24.99 kg/m^2^), normal weight (24.99–18.50 kg/m^2^), and underweight (< 18.50 kg/m^2^). Self-rated health was measured with the first question of the SF36 and dichotomized into very good, good and moderate, or bad and very bad [29].

Health behavior was assessed with four dichotomized proxies including poor dietary habit (less than 2 pieces of fruit or vegetables per month), sedentary lifestyle (more than half of the day sitting), alcohol binge drinking (more than one bottle of wine or equivalent per week) and current tobacco use (cigarettes or other tobacco products).

Social stress was measured by four different proxies consisting of yes and no questions. Confirmatory answers to questions on the living situation (living alone), social support (lack thereof), social participation (social inactivity) and trust in the neighbors (distrusting their neighbor) served as indicators for social stress [5].

### Statistical analyses

The prevalence of GSs was presented for the entire study sample and stratified by age and sex. Chi-square tests were performed for comparison between the stratified groups. Bivariate analyses were conducted examining the relationship between health status, health behavior and social stress. Due to high correlations among health status indicators, only the presence of a chronic condition was used in adjustments.

The associations between different measures of social position and presence of a GS were examined using binomial logistic regression (odds ratios [ORs] and 95% confidence interval [CI]). First, all analyses were adjusted for age and sex. In a next step, the analyses were further adjusted for chronic conditions (model 1), health behavior (model 2), chronic conditions and health-behavior (model 3), and finally chronic conditions, health behavior and social stress (model 4).

Missing information on covariates was treated as a separate category in the analysis. Missing information ranged mainly from 0.1 to 6.0%. However, data on level of education, tobacco use and alcohol use had missing information of 12.3, 13.4 and 12.3% respectively.

IBM SPSS Statistics 24 for Windows (IBM SPSS Inc., Chicago, Illinois, USA) was used for all statistical analyses.

## Results

In total, 12,333 (70.0%) older community-dwellers reported at least one GS. Table 1 shows insomnia as the most commonly reported GS (39.1%). GSs were more prevalent among women, except for severe hearing loss (men 22.3% vs. women 19.6%). Further, a higher prevalence of GSs was found among older community-dwellers with obesity or underweight, and those indicating to have a chronic condition (Table 2). GSs were particularly prevalent among those who rated their health as bad or very bad (*n* = 1199, 97.9%).

**Table 1:** Prevalence (%) of geriatric syndrome; by sex and age groups (*n* = 17,612)

Type of GS	Total	Sex			Age group (years)				
		Male *n* = 8117	Female *n* = 9495	*p* value^a^	65–69 *n* = 6248	70–74 *n* = 5130	75–79 *n* = 3617	80–84 *n* = 2617	*p* value^a^
Insomnia	39.1	30.4	46.6	< 0.01	36.8	40.2	40.4	40.7	< 0.01
Incontinence	26.4	19.2	32.6	< 0.01	20.5	25.8	29.7	37.1	< 0.01
Severe hearing problem	20.8	22.3	19.6	< 0.01	19.0	19.9	21.9	25.6	< 0.01
Functional decline	20.4	16.2	23.9	< 0.01	11.1	16.9	26.1	41.2	< 0.01
Fall	10.5	8.9	12.0	< 0.01	7.0	10.2	12.2	17.3	< 0.01
Severe vision problem	4.2	3.8	4.6	< 0.01	2.6	3.7	4.7	8.5	< 0.01
Depressive disorder	1.9	1.5	2.3	< 0.01	1.5	1.7	2.4	2.8	< 0.01
At least one geriatric syndrome	70.0	62.4	76.5	< 0.01	62.3	69.2	75.2	83.0	< 0.01

*Note*

^a^ *p*-value of Pearson Chi-square tests for comparison of distribution

**Table 2:** Prevalence and 95% CI of geriatric syndrome by different characteristics of the study population (*n* = 17,612)

Characteristics	Category	Geriatric syndrome^a^	
		n	%
*Health status*			
Self-rated health	Very good, good and moderate	10,834	67.7
	Bad and very bad	1199	97.9
BMI status	Obese	1771	78.6
	Overweight	4654	68.8
	Normal	5181	67.5
	Underweight	210	76.1
Chronic conditions	No	4315	63.0
	Yes	7827	74.3
*Health behavior*			
Poor dietary habit	No	10,431	68.8
	Yes	1066	74.6
Sedentary lifestyle	No	9943	67.2
	Yes	1949	86.4
Alcohol binge drinking	No	9540	68.4
	Yes	986	74.5
Current Tobacco user	No	9073	69.7
	Yes	1713	70,7
*Social stress*			
Living alone	No	7737	66.3
	Yes	4493	77.2
Lack of social support	No	10,528	68.8
	Yes	1495	77.8
Socially inactive	No	6321	66.5
	Yes	5310	74.1
Distrust in neighborhood	No	11,342	69.2
	Yes	739	80.6

*Note*

^a^ Reported at least one geriatric syndrome

Among the measures of social position, GS were most prevalent among older people with financial stress (87.7%) (Table 3). All measures of social position were significantly associated with presence of GS (Table 3), when adjusting only for age and sex. Financial stress showed the strongest association with GSs (OR, 3.33; 95% CI, 2.75–4.03).

**Table 3:** Prevalence and odds rations (95% CI) for geriatric syndromes by measures of social position (n = 17,612)

Characteristics	Category	Geriatric syndromes ^a^				
		n	%		Odds ratio	95% CI ^b^
Age (in years)	65–69	3892	62.3		1.00	
	70–74	3549	69.2		1.37	1.27–1.49
	75–79	2721	75.2		1.81	1.65–1.99
	80–84	2171	83.0		2.89	2.58–3.24
Sex	Male	5069	62.4		1.00	
	Female	7264	76.5		1.93	1.80–2.06
Civil status	Married	6817	66.5		1.00	
	Unmarried	912	70.3		1.23	1.08–1.40
	Divorced	2343	72.7		1.26	1.15–1.38
	Widowed	2257	79.5		1.29	1.16–1.43
Country of origin	Sweden	10,128	68.8		1.00	
	Other Nordic countries	1002	74.7		1.26	1.11–1.44
	Other European	826	76.6		1.53	1.32–1.77
	Rest of the world	377	79.4		1.87	1.48–2.35
Highest level of education	University education	3373	65.8		1.00	
	Upper secondary school	4351	69.0		1.15	1.06–1.24
	Primary school (≤ 9y)	2885	71.6		1.22	1.11–1.33
Type of Housing	Own accommodation	8620	67.2		1.00	
	Rented accommodation	3209	76.4		1.43	1.32–1.55
	Other	374	83.9		2.24	1.73–2.91
Financial Stress - General	No	11,104	68.7		1.00	
	Yes	904	87.7		3.33	2.75–4.03

*Note*

^a^ Reported at least one GS

^b^ Adjusted for sex and age

Financial stress remained strongly associated with GSs after adjustments for health status, i.e. chronic conditions and health behavior. Primary and secondary school education, and unmarried older people did not remain associated with GSs (Table 4) after taking chronic conditions and health behavior into account. Additional adjustment for social stress attenuated the associations of social position with GSs, but remained statistical significant, except for older people born outside the Nordic countries. Financial stress was most strongly associated with the presence of GSs even in the adjusted models (adj. OR, 2.59; 95% CI, 2.13–3.15). Financial stress was also associated with most types of geriatric syndromes (Additional file 2: Table S1.).

**Table 4:** Association between social position and geriatric syndromes, adjusted odd ratios and 95% CI

	Model 1	Model 2	Model 3	Model 4
Civil status				
Married	1.00	1.00	1.00	1.00
Unmarried	1.25 (1.10–1.43)	1.11 (0.97–1.27)	1.14 (1.00–1.30)	1.06 (0.93–1.20)
Divorced	1.26 (1.15–1.38)	1.16 (1.06–1.27)	1.16 (1.06–1.27)	1.11 (1.01–1.22)
Widowed	1.27 (1.14–1.42)	1.22 (1.10–1.36)	1.21 (1.08–1.35)	1.19 (1.07–1.22)
Country of origin				
Sweden	1.00	1.00	1.00	1.00
Other Nordic countries	1.23 (1.07–1.40)	1.20 (1.05–1.38)	1.18 (1.03–1.35)	1.12 (0.97–1.28)
Other European	1.51 (1.30–1.75)	1.48 (1.27–1.72)	1.47 (1.26–1.71)	1.39 (1.19–1.62)
Rest of the world	1.83 (1.45–2.31)	1.65 (1.30–2.09)	1.63 (1.29–2.08)	1.49 (1.17–1.90)
Highest level of education				
University education	1.00	1.00	1.00	1.00
Upper secondary school	1.11 (1.02–1.20)	1.12 (1.03–1.22)	1.09 (1.00–1.18)	1.05 (0.97–1.15)
Primary school (≤ 9y)	1.16 (1.05–1.27)	1.14 (1.04–1.25)	1.09 (0.99–1.20)	1.02 (0.93–1.13)
Type of housing				
Own accommodation	1.00	1.00	1.00	1.00
Rented accommodation	1.40 (1.29–1.52)	1.30 (1.19–1.41)	1.28 (1.17–1.39)	1.19 (1.10–1.30)
Other	2.27 (1.74–2.96)	1.72 (1.32–2.25)	1.78 (1.36–2.33)	1.67 (1.28–2.20)
Financial stress - General				
No	1.00	1.00	1.00	1.00
Yes	3.22 (2.65–3.90)	2.89 (2.38–3.51)	2.84 (2.33–3.45)	2.59 (2.13–3.15)

Note:

Model 1: adjusted by age, sex and chronic conditions

Model 2: adjusted by age, sex and health behaviors

Model 3: adjusted by age, sex, chronic conditions and health behaviors

Model 4: adjusted by age, sex, chronic condition, health behavior and social stressors (excl. Living alone)

## Discussion

Our study shows that GSs were highly prevelant (70.0%) among older community-dwellers in Stockholm County. The observerd prevalance varied acrossdifferent GSs. Older community-dwellers with self-reported financial stress, those not owning housing, as well as those that were widowed had the highest prevalence of geriatric syndromes. Presence of GSs was associated with all five measures of social position i.e. civil status, country of origin, level of education, type of housing and financial stress even after adjustment for age, sex and health status (i.e. chronic conditions). These associations between social position measures and geriatric syndromes remained after additional adjustments for health behavior and social stress, except for measures like education, i.e. primary and seconday school education, civil status, i.e. being unmarried and country of origin, i.e. being born outside of the nordic countries. Financial stress was by far most strongly associated with GSs. Those reporting financial stress were more than twice as common to experience GSs than those who did not.

All GSs and all participants aggregated, the high prevalence we observed compares to some extent to that reported in previous population-based studies on GSs [12, 17, 18], with estimations up to 49.9% [18],76.3% [17] and 80.5% [12], compared to our 70.0%. The difference between studies can be a reflection of differences in either the specific GSs combined, e.g. inclusion of polypharmacy [12] or the population groups, some focusing on female community-dwellers e.g. Women’s Health Initiative Observational Study [17], people aged 75 years and older [12] or community-dwellers and nursing home residents [18]. Our study includes seven of the most common GSs [10, 17, 18, 24, 30].

In regard to specific types of GSs, varying prevalences are also noted between our results and other studies [31, 32], including vision and hearing impairment [18] and falls [17]. However, prevalences vary for some other specific GSs, e.g. the prevalence of urinary incontinence was 29.3% [17] and 9.3% [18] compared to our 26.4%, which partly can be explained by the use of different definitions e.g. use of pads [18] vs. presence of urinary leakage.

When it comes to differences across social measures, it is of note that earlier studies have also shown an association between financial stress and age-dependent health conditions [4, 8, 13, 30, 33–35], but not yet to prevalent GSs in general, a potential indicator of upcoming new health conditions [10, 17, 18]. A previous study has shown a high prevalence of GSs among people in poverty [13], and studies on specific types of GSs have shown an increased risk with financial stress and also type of housing [34, 35]. A range of factors can explain the association between financial stress and GSs, among which health risk behavior [14], health status [1, 30] and living circumstances [35, 36]. But, it is also of note, that in our study, the association remains strong after taking all these factors into account.

The fact that older people born outside the Nordic countries have higher odds for GSs echoes earlier studies showing poorer health and higher prevalence of GSs in this population group [23, 37]. While we adjusted for contributing factors like social stressors [37], it remains unclear whether the association is a matter of higher physical vulnerability [37], less use of in-hospital care due to difference in illness presention, health illiteracy or communicaion barriers [38] or both. This population group may require particular attention, as prevention of GSs may decrease the risk of new chronic conditions and disability [2, 10, 18].

Poorer health among single persons, divorced and widowed, has been demonstrated in various studies [1, 5, 15]. In our study we also observe a significant association with GSs even after adjustments for chronic conditions, health behavior and social stressors (excluding living alone to avoid overadjustment when analysing civil status).

To date, study results on the association between education and GSs are mixed [1, 5, 13, 18]. We find a significant association between education and GSs in the crude analyses, but not in the adjusted ones suggests that health status, health behavior and social stress contribute largely to explain the association. With the majority of older people, in this study, holding a primary school degree as their highest educational attaintment, the low level of discrimination between levels of education may also affect the results [39].

Besides the large sample size, another strengths were the availability of different self-reported geriatrics syndromes in this population of older swedish community-dwellers from the Stockholm County Council, Public Health Survey. The same protocol was applied throughout the different survey years [24]. While self-reported data is prone to misclassification, it is more suited to capture the prevalence of GSs among older community-dwellers compared to registry information as many GSs are normally not officially coded in registers [40]. While other health surveys only capture some GS, the Stockholm County Council Public Health Survey is quite unique as the questions identify all specific GSs commonly reported in other GSs studies [10, 12, 23, 31, 32].

However, self-reports on GSs are also prone to underreporting, as older people may be less likely to report undesirable conditions [41], or consider them as “normal” age-related conditions. It is difficult to asses the affect of underreporting on our results, but we may have underestimated the magnitude of specific single GSs.

Further limitations concern non-participation bias in the survey. Very disabled older community-dwellers may not be fit enough to respond to the survey due to physical or cognitive impairments leading to an underestimation of the prevalence of GSs. This may weaken the external validity of our study and also for example older people born outside the Nordic countries are underpresented among the participants [24]. Despite lower response-rate among some subgroups, in terms of morbidity the Stockholm County Council Public Health survey data has been shown to be generally comparable to that of the Swedish population aged 65–80 years [42]. Those aged 80 years or older tended to be even slightly healthier than the population [42].

Our study does not allow any conclusions on causality or trajectories, as it investigates cross-sectional associations in terms of prevalence. However, some measures for social position like level of education that are stable over time, especially at the age of 65 and above, can be assumed to precede single or a set of GSs. Yet for other meassures like financial stress and type of housing a potential reverse causality could exist. A longitudinal study would be warranted to disentangle these effects and assess the risks of low social position in developing GSs.

## Conclusion

In this large population-based study, there is a high prevalence of GSs among older Swedish community-dwellers. Lower social position, especially the presence of financial stress, was associated with a higher risk of GSs independent of health behaviour and health status. More attention is required on social position when adressing,delaying or even preventing GSs among older community-dwellers.

## Supplementary information

**Additional file 1.** Additional information on study design and sample.

**Additional file 2: Table S1.** Showing the association between social position and number and specific types of geriatric syndromes, adjusted by age, sex, chronic condition, health behavior, social stressors (excl. living alone).

## Data Availability

The datasets used and/or analysed during the current study are available from the corresponding author on reasonable request.

## Abbreviations

BMI: Body mass index
GS: Geriatric syndrome
LISA: Database for health insurance and labor market studies
