# Supplementary material for: Social position and geriatric syndromes among Swedish older people: a population-based study
Source: BMC Geriatr. 2019 Oct 15;19:267. doi: 10.1186/s12877-019-1295-8 (PMC6792184; doi:10.1186/s12877-019-1295-8)
Supplement: Supplementary file 1 — Additional file 1. Additional information on study design and sample. [file 12877_2019_1295_MOESM1_ESM.docx]

**Supplementary Material - methods**

Study design and sample

Data was drawn from three cross-sectional Stockholm County Council Public Health Surveys (i.e. 2006, 2010, and 2014). The Public Health Surveys were conducted every four years among approximately 50,000 randomly selected individuals aged 18-84 years (from 2010, individuals above 84 years were also included) [24]. The selection was based on area-specific random sampling of individuals living in Stockholm County. The Stockholm County counts 1.9 million inhabitants representing 21% of the Swedish population. In the current study, the sample was restricted to people aged 65 to 84 years (n=18,592) with a final study population of 17,612 participants. Among those, the response rates were 74.5% (n=6,713), 74.1% (n=7,153) and 60.1% (n=4,726) in 2006, 2010 and 2014 respectively. Non-respondents among the invited individuals were more likely to be men, foreign-born, divorced or unmarried, unemployed and to represent the lower income quartile[24]. The postal questionnaires and web-based questionnaires were used for collecting data. . Subsequently, the data was linked with the LISA registers kept by Statistics Sweden and the National Board of Health and Welfare through the unique personal identity number of each individual [24].
