# Supplementary material for: Social position and geriatric syndromes among Swedish older people: a population-based study
Source: BMC Geriatr. 2019 Oct 15;19:267. doi: 10.1186/s12877-019-1295-8 (PMC6792184; doi:10.1186/s12877-019-1295-8)
Supplement: Supplementary file 2 — Additional file 2: Table S1. Showing the association between social position and number and specific types of geriatric syndromes, adjusted by age, sex, chronic condition, health behavior, social stressors (excl. living alone). [file 12877_2019_1295_MOESM2_ESM.docx]

Supple. Table 1. Showing the association between social position and number and specific types of geriatric syndromes, adjusted by age, sex, chronic condition, health behavior, social stressors (excl. living alone).

| **Suppl. Table 1.** Association between social position and specific geriatric syndromes, adjusted odd ratios and 95% CI. | | | | | | | | |
| --- | --- | --- | --- | --- | --- | --- | --- | --- |
|  | | **Specific types of geriatric syndromes^a^** | | | | | | |
|  | | **Insomnia** | **Incontinence** | **Severe hearing problem** | **Functional decline** | **Fall** | **Severe vision problem** | **Depressive disorder** |
| Civil status | |  |  |  |  |  |  |  |
|  | Married | REF | REF | REF | REF | REF | REF | REF |
|  | Unmarried | 0.98 (0.86 – 1.11) | 1.02 (0.88 – 1.17) | 0.93 (0.80 – 1.08) | 1.11 (0.93 – 1.31) | 1.17 (0.97 – 1.42) | 1.05 (0.77 – 1.41) | 1.38 (0.94 – 2.00) |
|  | Divorced | 1.08 (0.99 – 1.18) | 1.03 (0.94 – 1.13) | 1.04 (0.94 – 1.15) | 1.19 (1.06 – 1.33) | 1.21 (1.06 – 1.39) | 1.24 (1.02 – 1.52) | 1.11 (0.82 – 1.50) |
|  | Widowed | 1.02 (0.92 – 1.12) | 1.11 (1.01 – 1.22) | 1.12 (1.01 – 1.25) | 1.19 (1.06 – 1.34) | 1.10 (0.96 – 1.27) | 1.08 (0.88 - 1.34) | 1.36 (1.01 – 1.85) |
| Country of origin | |  |  |  |  |  |  |  |
|  | Sweden | REF | REF | REF | REF | REF | REF | REF |
|  | Other Nordic countries | 1.24 (1.10 – 1.40) | 0.92 (0.80 – 1.05) | 1.08 (0.94 – 1.24) | 1.04 (0.89 – 1.21) | 0.91 (0.75 – 1.10) | 1.22 (0.94 – 1.60) | 1.04 (0.71 – 1.53) |
|  | Other European | 1.61 (1.41 – 1.83) | 0.86 (0.74 – 0.99) | 1.06 (0.91 – 1.23) | 1.02 (0.86 – 1.22) | 1.21 (1.01 – 1.48) | 1.18 (0.88 – 1.57) | 1.32 (0.90 – 1.93) |
|  | Rest of the world | 1.58 (1.29 – 1.93) | 1.28 (1.04 – 1.57) | 1.13 (0.91 – 1.41) | 1.61 (1.27 – 2.05) | 1.50 (1.16 – 1.95) | 2.20 (1.58 – 3.05) | 1.16 (0.68 – 1.99) |
| Highest level of education | |  |  |  |  |  |  |  |
|  | University education | REF | REF | REF | REF | REF | REF | REF |
|  | Upper secondary school | 1.03 (0.95 – 1.12) | 0.93 (0.85 – 1.01) | 1.09 (0.99 – 1.20) | 1.42 (1.27 – 1.61) | 0.91 (0.80 – 1.03) | 1.07 (0.86 – 1.33) | 0.85 (0.64 – 1.13) |
|  | Primary school (≤ 9y) | 0.96 (0.87 – 1.05) | 0.94 (0.85 – 1.04) | 1.15 (1.03 – 1.28) | 1.59 (1.40 – 1.81) | 0.85 (0.74 – 0.99) | 1.13 (0.90 – 1.43) | 0.65 (0.47 – 0.91) |
| Type of housing | |  |  |  |  |  |  |  |
|  | Own accommodation | REF | REF | REF | REF | REF | REF | REF |
|  | Rented accommodation | 1.07 (0.99 – 1.16) | 0.98 (0.90 – 1.06) | 1.06 (0.97 – 1.16) | 1.39 (1.26 – 1.54) | 1.03 (0.92 – 1.16) | 1.40 (1.17 – 1.66) | 1.01 (0.78 – 1.32) |
|  | Other | 1.27 (1.03 – 1.57) | 1.89 (1.54 – 2.33) | 1.19 (0.95 – 1.49) | 2.50 (1.96 – 3.18) | 1.69 (1.31 – 2.18) | 2.89 (2.14 – 3.91) | 2.66 (1.76 – 4.00) |
| Financial stress - General | |  |  |  |  |  |  |  |
|  | No | REF | REF | REF | REF | REF | REF | REF |
|  | Yes | 1.61 (1.40 – 1.85) | 1.72 (1.49 – 1.98) | 1.10 (0.94 – 1.29) | 2.30 (1.97 – 2.69) | 1.88 (1.59 – 2.24) | 1.92 (1.51 – 2.50) | 2.34 (1.72 – 3.18) |
| Note:  Bold letters indicate a statistical significant association *p-value* <0.05  ^a^ adjusted by age, sex, chronic condition, health behavior and social stressors (excl. living alone) | | | | | | | | |
